# Using Modified Antigenic Sequences to Develop Cancer Vaccines: Are We Losing the Focus?

**DOI:** 10.1371/journal.pmed.0010026

**Published:** 2004-11-30

**Authors:** Danila Valmori, Maha Ayyoub

## Abstract

Are short synthetic peptides the key to developing cancer vaccines? And what are the obstacles in the way?

## Tumor Antigen Recognition by Cytolytic T Lymphocytes

CD8^+^ cytolytic T lymphocytes (CTLs) are the primary effector cells of the adaptive immune system and have a major role in protecting us from a vast array of diseases including cancer. CTLs specifically recognize and lyse targets through the interaction of T cell receptors (TCRs) on the surface of the T lymphocyte with protein fragments (peptides) presented on the surface of target cells, in association with major histocompatibility complex (MHC) class I molecules. When a particular CTL interacts with a target cell, it rapidly divides to form a clonal population of T cells with the identical TCR.

Townsend and colleagues first elucidated the molecular basis of target cell recognition by CTLs in 1985 [1]. They showed that antigens are processed inside the target cell into nine- or ten-amino-acid-long peptides, which are then presented at the surface in association with MHC class I molecules. This discovery suggested the possibility of using short synthetic peptides mimicking naturally processed antigens as immunotherapeutic drugs and vaccines. Short synthetic peptides are ideal for drug development because of the relatively low cost of production, easy storage, and high safety. However, not all peptides and MHC alleles work well together to stimulate CTLs. So for clinical use, either patients would have to be selected for treatment based on their MHC I type or it would be necessary to make multiple peptides to cover the majority of MHC class I alleles in a given population.

Furthermore, before Boon and colleagues cloned the first antigen recognized by tumor-reactive CTLs in 1991 [2], it was not clear which antigens were recognized by tumor-reactive CTLs in humans; so, it was not possible to rationally design cancer vaccines. Now, however, a long list of more or less tumor-specific antigens has been generated [3]. Most of the peptides identified so far are either normal self proteins aberrantly expressed in cancer but not in most other adult normal tissues or tissue-specific antigens also expressed in certain types of cancer. Some patients show a spontaneous CD8^+^ T cell response (occasionally at high levels) that is specific for several of these antigens. The development of such responses, however, requires a large tumor load, occurs late in the disease, and probably does not cause the efficient destruction of the tumor cells [4]. Thus, a central objective in cancer immunotherapy is to efficiently produce tumor-reactive CTLs at an earlier phase of the disease.

## Heteroclitic Tumor Antigen Peptides

Unfortunately, some synthetic peptides, including some corresponding to immunodominant epitopes (those which cause the biggest part of the immune response) from tumor antigens, only seem to bind MHC class I molecules with medium to low affinity and/or are recognized by specific T cells with relatively low avidity. These characteristics are the likely cause of the poor immune reaction generated by these peptides [5]. One strategy to improve the immune reaction is to make what are called heteroclitic antigen variants. By improving either peptide binding to MHC, recognition by TCRs, or both, these variants have increased peptide antigenicity and immunogenicity.

Solinger and colleagues were the first to describe antigen variants producing T cell responses that were stronger than those elicited by the parental sequences [6]. Some heteroclitic tumor antigen peptides that showed highly improved antigenicity and immunogenicity in preclinical studies, and which also cross-reacted well with CTLs generated against the parental sequence, were tested in clinical trials. The peptides selected for trials mostly contained substitutions of anchoring amino acids that were designed to increase peptide binding to the MHC molecule while minimally changing the shape of the epitope [7,8].

**Figure 1:**
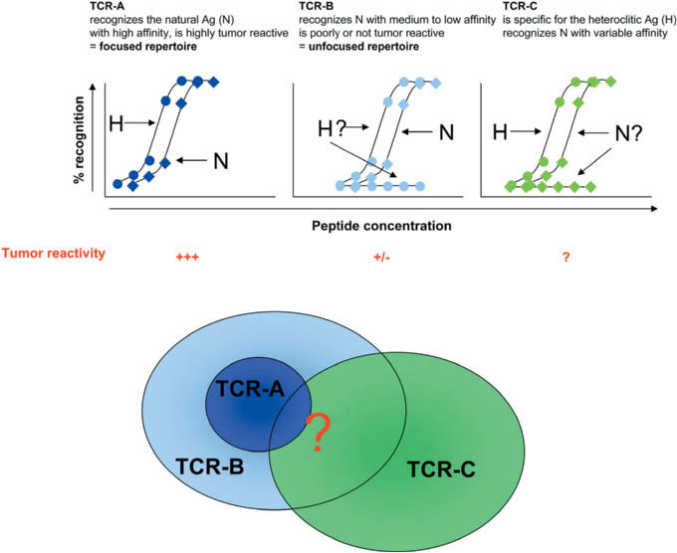
Synthetic Peptides Used for Vaccination May Fail to Faithfully Mimic the Naturally Processed Antigens The TCR repertoire specific for the natural tumor antigen (N) contains a group of CD8^+^ T cells that recognize N with high functional avidity and display high tumor reactivity (TCR-A, focused). TCR-A is stimulated by the natural ligand and expands during spontaneous responses to the tumor in some patients with antigen-expressing tumors. A larger group of CD8^+^ T cells able to recognize N also exists in the naïve T cell repertoire (TCR-B). TCR-B recognizes N with decreased avidity and shows low to undetectable tumor reactivity (unfocused). Heteroclitic peptides (H) are analogs of N that contain modifications that increase their immunogenicity. They are selected on the basis of their increased recognition by T cells from the TCR-A group. When used as immunogens, they elicit a group of CD8^+^ T cells specific for H (TCR-C). The reactivity of TCR-C will be variable and will depend on the extent of overlapping between the TCR-A, -B, and -C groups. In Lee and colleagues' study [9], most of the elicited CD8^+^ T cells belong to the TCR-B group (unfocused), suggesting a large overlap between TCR-C and TCR-B and a more limited overlap of TCR-C with TCR-A. This phenomenon can be explained by the structural difference between N and H. Other factors that may differ between natural and peptide-induced immune responses, including the density of the peptide on antigen-presenting cells and the mode of presentation (i.e., the nature of antigen-presenting cells), could also contribute to the outcome.

In a study by Lee and colleagues in this issue of *PLoS Medicine* [9], despite the careful study design, vaccination with these peptides resulted in the recruitment of T cells that bound antigens less efficiently and had lower tumor reactivity than those from the endogenous response to the tumor. The authors propose that the cause for the decreased affinity of vaccine-elicited CTLs could be the high antigen density of these synthetic peptides on antigen-presenting cells. An alternative explanation, however, is that the synthetic peptides used for vaccination simply fail to faithfully mimic the naturally processed antigens (Figure 1). The use of peptides that differ from those resulting from natural intracellular processing has previously given rise to similar problems [10,11]. In any case, the enormous diversity in the normal TCR repertoire provides a molecular explanation of the observed phenomenon. These results emphasize how difficult it is to translate findings, such as the spectacular results obtained by the vaccination of TCR transgenic mice with heteroclitic peptides [12], into an application for normal animals and humans.

## Conclusion

It is increasingly clear that even the smallest alteration in the structure of the MHC peptide complex can result in significant changes in which TCRs are selected after vaccination. Thus, manipulating the immune T cell repertoire in vivo through the use of heteroclitic tumor antigen peptide variants could be harder than anticipated. As the field moves rapidly towards the use of new vaccine adjuvants with high immunogenic potential [13], reassessment of the immunogenicity of natural sequences could be worthwhile in some cases. In addition, the careful analysis of antigen-specific T cell clones, such as that reported here by Lee and colleagues, will be crucial to ascertain the quality of the elicited immune response.

## Abbreviations

CTL: CD8^+^ cytolytic T lymphocyte
MHC: major histocompatibility complex
TCR: T cell receptor
